# New physiological bench test reproducing nocturnal breathing pattern of patients with sleep disordered breathing

**DOI:** 10.1371/journal.pone.0225766

**Published:** 2019-12-05

**Authors:** Shuo Liu, Yann Rétory, Amélie Sagniez, Sébastien Hardy, François Cottin, Gabriel Roisman, Michel Petitjean

**Affiliations:** 1 Centre EXPLOR, Air Liquide Healthcare, Gentilly, France; 2 CIAMS, Univ. Paris-Sud, Université Paris-Saclay, Orsay Cedex, France; 3 CIAMS, Université d’Orléans, Orléans, France; 4 Centre du Sommeil, Service d’Explorations Fonctionnelles Multidisciplinaires, Hôpital Antoine Béclère, Assistance Publique-Hôpitaux de Paris, Clamart, France; Brigham and Women’s Hospital and Harvard Medical School, UNITED STATES

## Abstract

Previous studies have shown that Automatic Positive Airway Pressure devices display different behaviors when connected to a bench using theoretical respiratory cycle scripts. However, these scripts are limited and do not simulate physiological behavior during the night. Our aim was to develop a physiological bench that is able to simulate patient breathing airflow by integrating polygraph data. We developed an algorithm analyzing polygraph data and transformed this information into digital inputs required by the bench hardware to reproduce a patient breathing profile on bench. The inputs are respectively the simulated respiratory muscular effort pressure input for an artificial lung and the sealed chamber pressure to regulate the Starling resistor. We did simulations on our bench for a total of 8 hours and 59 minutes for a breathing profile from the demonstration recording of a Nox T3 Sleep Monitor. The simulation performance results showed that in terms of relative peak-valley amplitude of each breathing cycle, simulated bench airflow was biased by only 1.48% ± 6.80% compared to estimated polygraph nasal airflow for a total of 6,479 breathing cycles. For total respiratory cycle time, the average bias ± one standard deviation was 0.000 ± 0.288 seconds. For patient apnea events, our bench simulation had a sensitivity of 84.7% and a positive predictive value equal to 90.3%, considering 149 apneas detected both in polygraph nasal simulated bench airflows. Our new physiological bench would allow personalizing APAP device selection to each patient by taking into account individual characteristics of a sleep breathing profile.

## Introduction

Obstructive sleep apnea (OSA) is a sleep disordered breathing (SDB), characterized by repetitive narrowing or closure of the upper airway during sleep [1]. This leads to intermittent arterial oxygen desaturations [2], and also to increased activations of the sympathetic nervous system, which may result in complications in cardiovascular diseases [3], as well as arterial hypertension [4]. Patients affected by OSA have symptoms like heavy snoring, excessive diurnal somnolence, and difficulty in concentration and memory, all of which significantly reduce their quality of life. Depending on severity, moderate and severe syndromes are largely treated by using continuous positive air pressure (CPAP) devices to maintain open airways, and prevent the occurrence of adverse breathing events during sleep at night. These treatments have proven to be efficient by drastically reducing the number of breathing events [5]. Moreover, they have a clearly beneficial impact on diurnal activities by significantly reducing sleepiness [5]. However, one of the key issues in treating a SDB patient with CPAP is the choices of medical device available on the market and of the ventilation mode (constant positive airway pressure or automatic positive airway pressure (APAP)). APAP mode relies on the use of a dedicated algorithm driving the response of the device to breathing events such as apneas or a significant reduction of respiratory flow. These algorithms are different from one device to another [6–13] and several setting options [14–16] can be chosen by users. Because the algorithms are protected by patents, they are like a black box to the public [17] and cannot be evaluated directly. Most of the time, device functioning can be retrospectively and indirectly observed by recording patient ventilation under APAP [18,19]. This is why there has been some work to develop respiratory benches, as reported in the literature [6–13], to evaluate miscellaneous devices. Some benches consist of a lung simulator to mimic the patient’s respiratory airflow [6]. Other benches are additionally connected to an upper airway simulator by use of either an obstruction valve [9,13] or a Starling resistor [7,8,10–12], whose resistances are conditioned to pressure changes from APAP devices in different manners. The mechanical impedance in an obstruction valve is controlled via a predefined computer program, in function of the instantaneous pressure of the APAP. On the contrary, the resistance of Starling resistor reacts mechanically and automatically to the pressure changes. Bench hardware can be programmed to simulate the artificially composed respiratory scenarios, which generally contain a string of repetitive disordered breathing events, i.e. obstructive and central apneas, obstructive and central hypopneas, inspiratory flow limitation, snoring. Various APAP devices are then connected to the bench simulation. Their pressure responses to the SDB events are recorded, assessed and compared. However, all these benches use airflow from a limited database of breathing airflow short segments recorded in patients and/or artificially designed. Thus, the respiratory scenarios simulated on bench cannot represent completely the physiological variability and chronology of human breathing. This is why it is difficult to generalize the bench-observed treatment efficiencies to one individual patient. Furthermore, as APAP manufacturers are adjusting their algorithm responses as a more and more detailed function of patient physiological breathing behaviors, this also underlines the needs to adapt the bench testing in a physiological manner. Based on these contexts, Isetta *et al*. mimicked a full night of one female OSA phenotype on their bench [13]. However, there still existed a distinction between bench simulated breathing profile and the one of a specific patient. Thus, a physiological bench, which could replicate automatically any apneic patient breathing profile by using its polygraph recordings, should solve this problem. Thus, the aim of our study was to develop a new approach for bench testing, which enables the automatic reproduction of a patient nasal breathing phenotype, taking into account the central and obstructive characteristics of each respiratory cycle. To accomplish this, the secondary aim was to develop an algorithm that is able to process a patient’s night polygraph data, and to calculate the digital inputs required to control the hardware of the bench simulation device.

## Material and methods

### Bench test system design

Our bench system is composed of two parts: signal processing and bench simulation. Our work mainly focused on designing an algorithm that is able to integrate polygraph data as inputs, and to return as outputs the necessary digital inputs to control our bench hardware. This hardware consists of an artificial lung using a piston to mimic patient pulmonary motion during respiration, driven by simulated respiratory muscular effort pressure (ΔP_mus_) and a Starling resistor that mimics upper airway obstruction by varying its resistance. Our algorithm calculates the level of upper airway obstruction as well as the pressure intensity of the respiratory muscular effort to be simulated in each breathing cycle of a patient by analyzing polygraph data in a way that is most in line with AASM rules [20].

**Fig 1:**
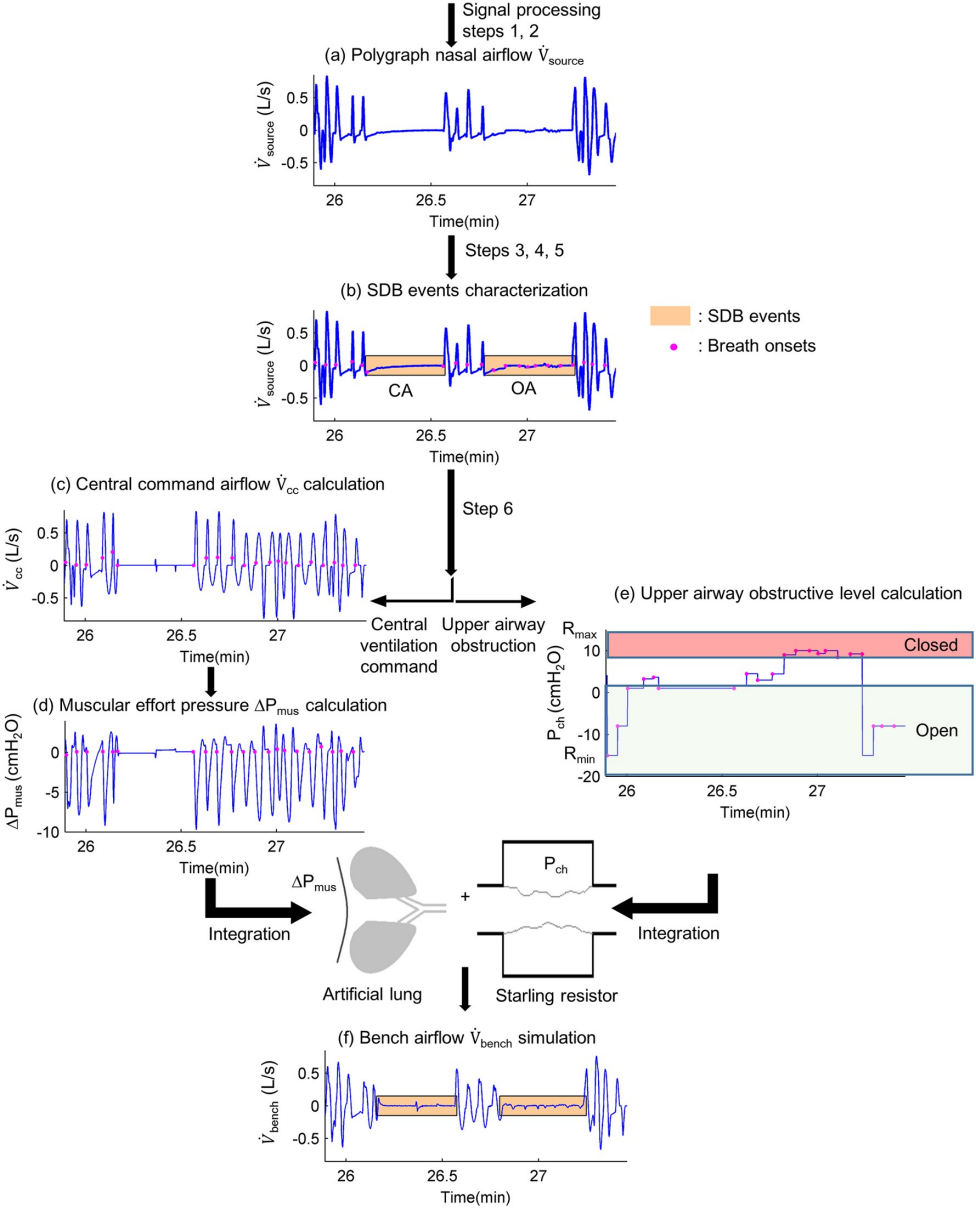
Signal processing overview from polygraph data to its breathing profile simulated on bench. The signal processing steps or steps refer to the main steps described in polygraph signal processing section. (a) Polygraph nasal airflow, steps 1 and 2: nasal airflow (${\dot{\text{V}}}_{\text{source}}$) of period without movement artifact is derived from polygraph raw signals. It represents the target breathing airflow, which is aimed to be simulated on bench. (b) Sleep disordered breathing events characterization, steps 3, 4 and 5: breathing onset positions (*) and sleep disordered breathing (SDB) events (orange frames) are identified. SDB events can be obstructive apneas (OA), central apneas (CA), obstructive hypopneas, central hypopneas. (c) Central command airflow calculation, step 6: the estimated non-obstructed nasal airflow issuing from central ventilation command (${\dot{\text{V}}}_{\text{cc}}$) is calculated by combining information of ${\dot{\text{V}}}_{\text{source}}$ and SDB events characterization. During normal breathing periods or central SDB events, ${\dot{\text{V}}}_{\text{cc}}$ is directly assumed identical to ${\dot{\text{V}}}_{\text{source}}$, whereas during obstructive breathing events, the amplitude of ${\dot{\text{V}}}_{\text{cc}}$ is hypothesized to be equal to ${\dot{\text{V}}}_{\text{source}}$ amplitude of the pre-event 2-minute baseline. (d) Muscular effort pressure calculation, step 6: the respiratory muscular effort pressure (ΔP_mus_) is calculated by taking into account ${\dot{\text{V}}}_{\text{cc}}$ as well as the lung compliance and airway resistance set in the active lung simulator. (e) Upper airway obstructive level calculation, step 6: the sealed chamber pressure (P_ch_) used to regulate Starling resistor for each breathing cycle is calculated as a function of ${\dot{\text{V}}}_{\text{cc}}$ and ${\dot{\text{V}}}_{\text{source}}$. (f) Bench airflow simulation: the bench-simulated airflow (${\dot{\text{V}}}_{\text{bench}}$) is obtained by controlling the active lung simulator and the Starling resistor with ΔP_mus_ and P_ch_.

Specifically, to simulate a targeted polygraph nasal airflow (${\dot{\text{V}}}_{\text{source}}$) on our bench (Fig 1), the resistance of a Starling resistor was increased during an obstructive respiratory event, and set to minimum during periods of normal breathing as well as central breathing events. The ΔP_mus_ used to command our artificial lung was calculated based on the breathing airflow issuing from a central ventilation command. We noted this estimated non-obstructed nasal airflow, without any obstruction occurring in the upper airways, as ${\dot{\text{V}}}_{\text{cc}}$.

### Bench hardware

**Fig 2:**
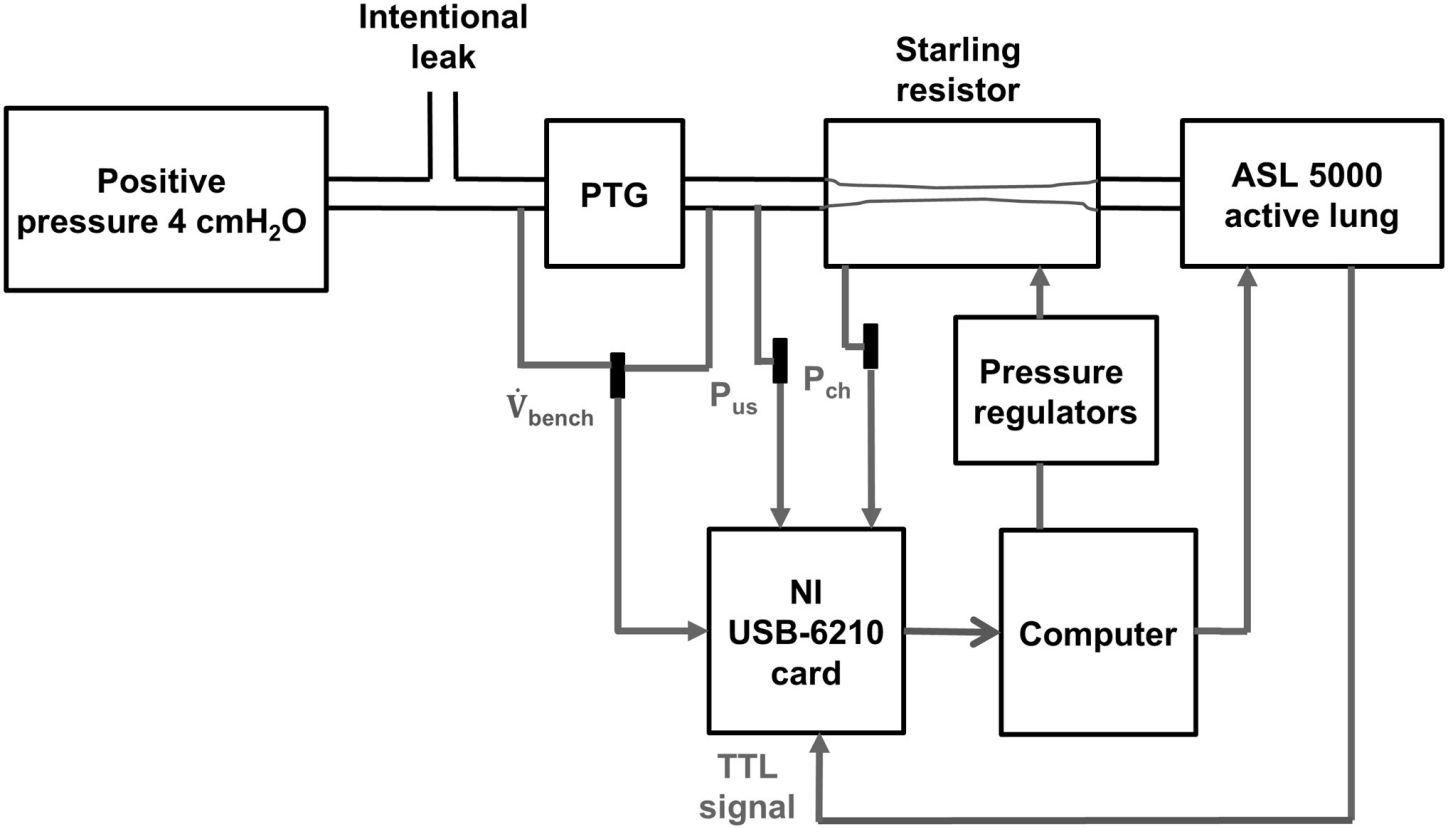
Bench hardware diagram. PTG: pneumotachograph; ${\dot{\text{V}}}_{\text{bench}}$: bench airflow measured by a pneumotachograph; P_us_: pressure at upstream of Starling resistor; P_ch_: sealed chamber pressure in Starling resistor; TTL signal: transistor-transistor logic signal.

As shown in Fig 2, the bench hardware used in our study to reproduce the polygraph breathing profile was derived from a setup described in a previous study from our laboratory [12]. The hardware consisted mainly of an active lung simulator ASL 5000 (IngMar Medical, Pittsburgh, USA) and a Starling resistor, in which a rubber tube could collapse as a function of transmural pressure. The transmural pressure is the difference between the intraluminal pressure at the upper stream (P_us_) and the extra-luminal pressure, which corresponds to the sealed chamber pressure (P_ch_). In detail, P_us_ was set to 4 cmH_2_O, as the minimal default pressure sent by APAP devices. Then, the obstructive state in the rubber tube of the Starling resistor was altered by varying P_ch_ via a pressure control system, which supplied continuous positive or negative pressures. The adjustment of obstruction level in the Starling resistor was triggered once at the beginning of each breathing cycle via a transistor-transistor logic (TTL) signal sent by the active lung simulator.

To simulate ventilatory function, we used a single-compartment model in the active lung simulator, in which a patient’s lung was modeled as a single compliance C and a single resistor R connected in series, representing respectively lung compliance and airway resistance with C = 80 mL/cmH_2_O and R = 7.25 cmH_2_O/(L/s). In our case, because we used a Starling resistor to mimic the upper airway pathophysiology, the airway resistance set in the active lung simulator did not take into account upper airway resistance. The digital input used to command each breathing cycle in active lung simulator was ΔP_mus_. There is a relationship between ΔP_mus_ and non-obstructed breathing airflow ${\dot{\text{V}}}_{\text{cc}}$, dominated by the equation

$$\Delta \text{Pmus}=-\frac{\text{Vcc}}{\text{C}}-\text{R}*\dot{V}cc,$$ (1)

in which V_cc_ represents pulmonary instantaneous volume, thus $Vcc=\int \dot{V}cc\;dt$.

The flow produced on the bench (${\dot{\text{V}}}_{\text{bench}}$) was monitored by a pneumotachograph located between the source of positive pressure 4 cmH_2_O and the Starling resistor (Fig 2).

All signals measured on the bench were sampled via a NI USB-6210 card (data acquisition card, National Instruments, USA) and a custom-developed LabVIEW (National Instruments) program at a rate of 20 Hz. Then, they were stored in a personal computer for further offline analysis.

### Polygraph signal processing

The polygraph data used in our study issued from a one-night demonstration recording by a Nox T3 Sleep Monitor (Nox Medical, Reykjavik, Iceland). On our bench, we reproduced a total of 8 hours and 59 minutes of this breathing profile by integrating polygraph recordings. The corresponding apnea-hypopnea index (AHI) of this recording was 22.6 events/h with apnea index (AI) = 15.5 events/h and hypopnea index (HI) = 7.1 events/h. The signals used in our study were respectively an acceleration signal sampled at 20 Hz, nasal pressure sampled at 200 Hz, thoracic and abdominal Respiratory Inductance Plethysmography (RIP thorax and RIP abdomen) sampled at 25 Hz, RIP flow at 25Hz, pulse oximetry (SpO_2_) sampled at 3 Hz, and audio volume sampled at 100 Hz.

**Fig 3:**
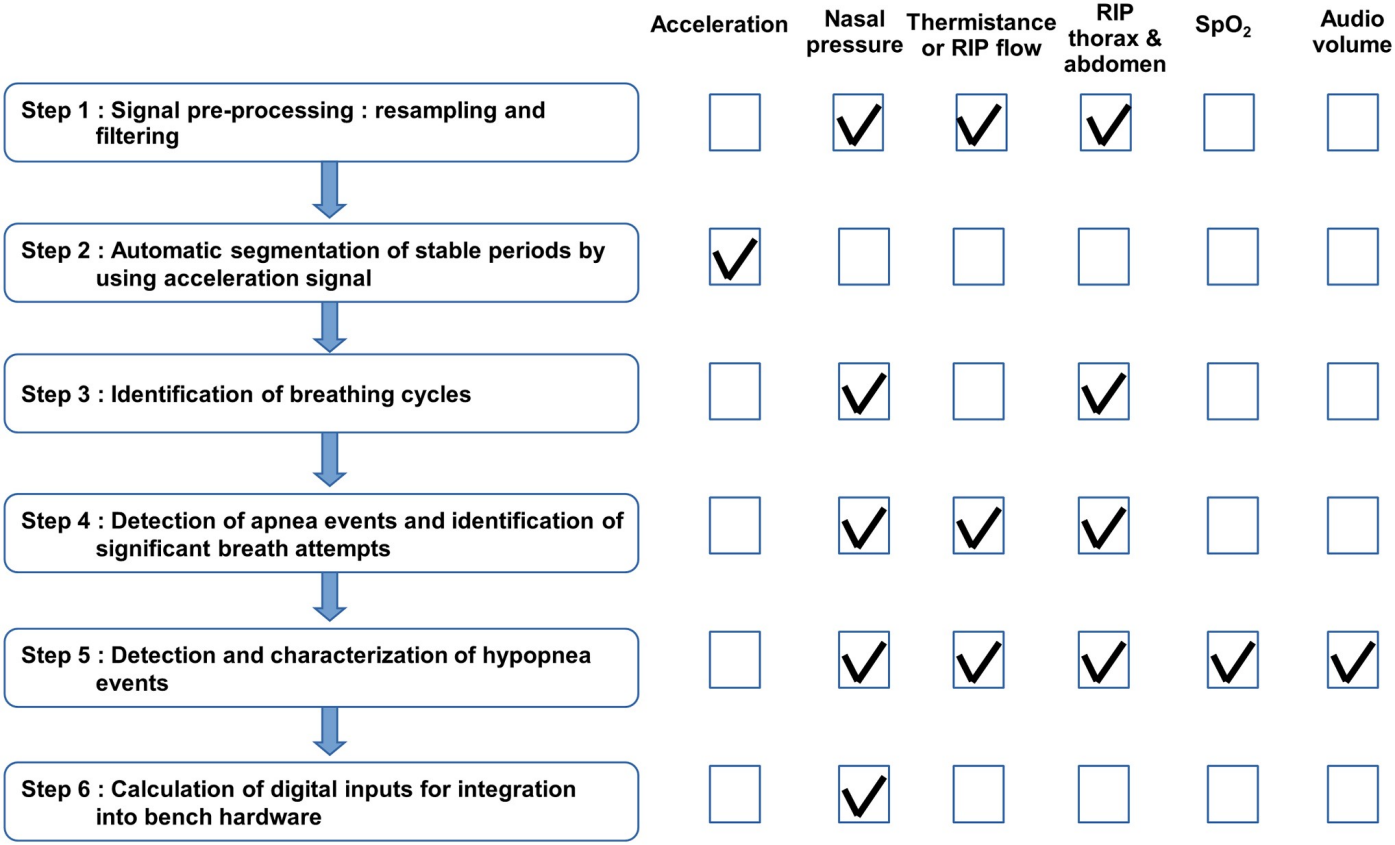
Main steps in polygraph signal processing. The checked signals next to each signal-processing step mean that they were directly associated with this step.

We developed an algorithm with Matlab (MATLAB, Signal Processing Toolbox and Statistics Toolbox Release 2013b, The MathWorks, Inc., Natick, Massachusetts, United States) which allowed for interpretation of polygraph signals and calculation of the digital inputs that needed to be integrated into the bench hardware. Concretely, the ΔP_mus_ to drive the artificial lung and the P_ch_ to regulate the obstructive level in the Starling resistor were used for bench simulation. Fig 3 describes the six main signal-processing steps implemented in the algorithm. Each step is then depicted in detail in the following subsections.

#### Step 1: Signal pre-processing: Resampling and filtering

Nasal pressure, RIP flow, audio volume, RIP thorax and abdomen were first down-sampled at 20 Hz to homogenize the whole signal data for further analyses. Then nasal pressure, RIP flow, RIP thorax and RIP abdomen were smoothed by a Savitzky-Golay filter [21]. In our conditions, the filter was constructed by fitting successive sub-sets of adjacent data points in 1 second with a third-degree polynomial function. According to AASM rules [20], the nasal airflow modulation with time ${\dot{\text{V}}}_{\text{source}}$ (20 Hz) was estimated as the square-root transformation of nasal pressure.

#### Step 2: Automatic segmentation of stable periods by using acceleration signal

**Fig 4:**
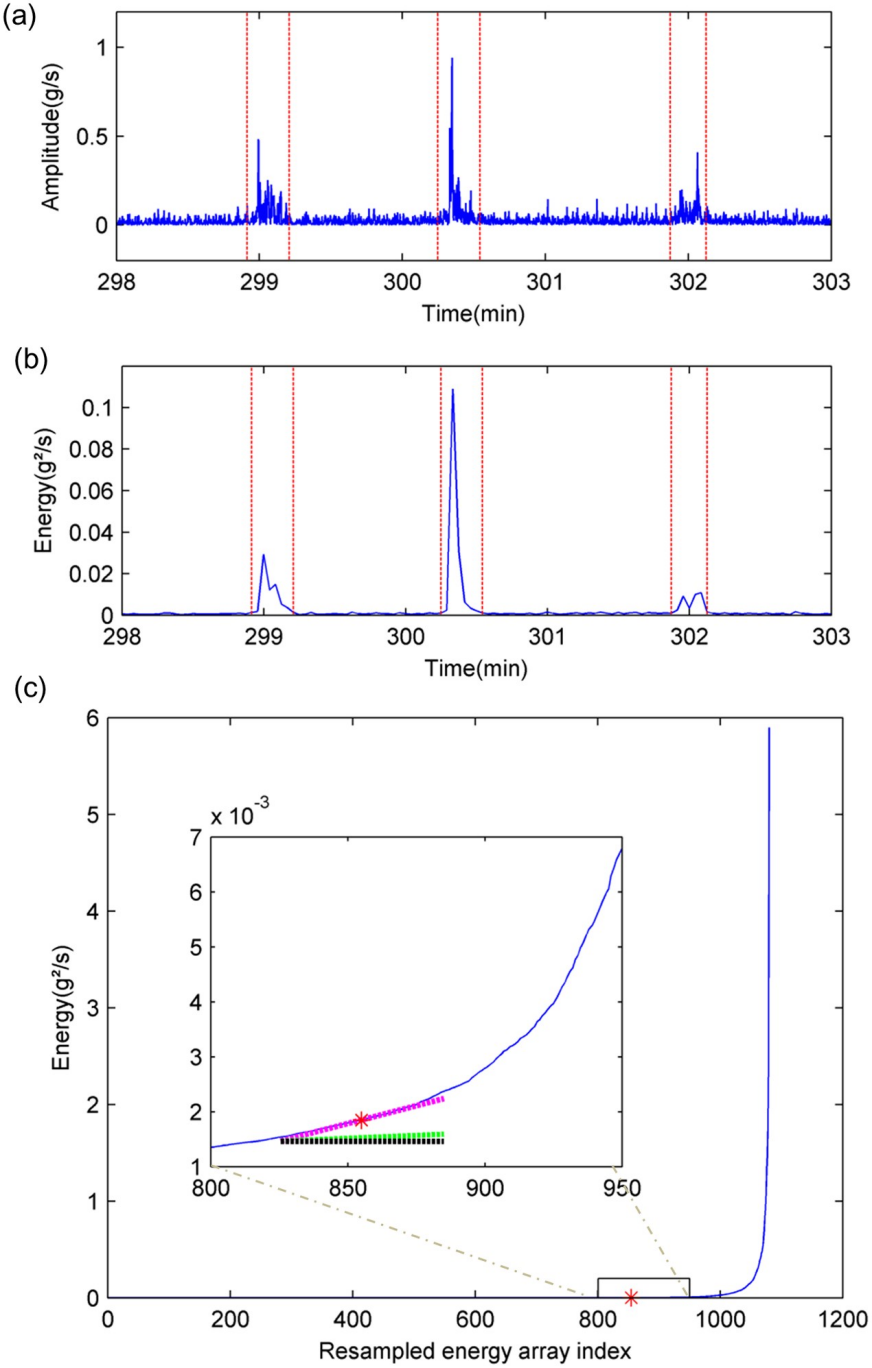
Segmentation of stable periods by analyzing acceleration signal. (a): Example of acceleration signal (20 Hz) obtained from polygraph. This signal corresponds to the Euclidean norm of three-axis accelerations measured by the accelerometer installed in the polygraph device. (b): Energy signal—calculated in frames of 5 seconds with 50% overlap. (c): Determination of frame energy threshold in the resampled increasing frame energy array, represented by the blue line. The magenta line is the current linear regression line, which fits into six consecutive points in the resampled increasing frame energy array. The green line’s slope is the average value of all previously calculated slopes. The black line is a reference horizontal line. The slope of the magenta line is greater than 5.8 times that of the green line. The energy value of the red asterisk corresponds to the median energy of the six consecutive points fitted by the magenta line. Thus, the amplitude of the red asterisk is determined as the frame energy threshold.

The aim of this second step was to avoid body motion artifacts in the respiratory signals. We chose to exclude periods with a high probability of artifact occurrence by using the 3D accelerometer signal (Fig 4(a)), which reflected body movement at night during recording. This signal was the calculation of the Euclidean norm of 3-axis acceleration.

Stable periods were characterized by low acceleration amplitude segments, in comparison to periods with movement in the 3D accelerometer signal. To automatically identify stable periods, our method was based on setting up an adaptive threshold in the energy signal of 3D acceleration. The energy signal (Fig 4(b)) was calculated in a frame of 5 seconds with an overlap of 50%, as shown in Eq (2), in which S_k_ (i) was the 3D accelerometer signal contained in the k^th^ frame of 100 data samples, and E_k_ represented the energy of the signal in the k^th^ frame.

$${\text{E}}_{\text{k}}=\sum_{i=1}^{100}|{\text{S}}_{\text{k}}\left(i\right){|}^{2}$$ (2)

To determine an appropriate energy threshold discriminating between high and low energy frames, the energy array was sorted by an ascending order in the first step. Then, in order to decrease the required computing power, the energy array was down-sampled to obtain a new energy array composed by 1080 samples (Fig 4(c)). To detect the change onset point in the sorted and down-sampled energy array, we chose to detect the slope change position. Thus, we calculated the slopes of every six consecutive energy points with a 50% overlap by performing a linear regression (Fig 4(c)). We obtained a slope array of 359 values. We assumed that the patient should spend at least 25% of the recording time sleeping with negligible body movement. Accordingly, the slope change position needed to be situated after index 90 (359*25% = 90) of the slope array. To find the slope change position, we divided each slope value situating from index 90 by the average of its previous slope values. If this ratio was greater than 5.8, a number that was empirically determined, the corresponding slope was then considered as the change position in slopes. Consequently, the energy threshold was determined as the median of the 6 consecutive energy values, from which the corresponding slope was calculated.

At the end, stable frames were combined if they overlapped and were lower than the calculated energy threshold. All considered signals in the remaining steps of the process were mostly extracted from these stable periods. Nevertheless, we allowed short periods of movement less than 2 minutes to maintain congruity of the polygraph signals.

#### Step 3: Identification of breathing cycles

**Fig 5:**
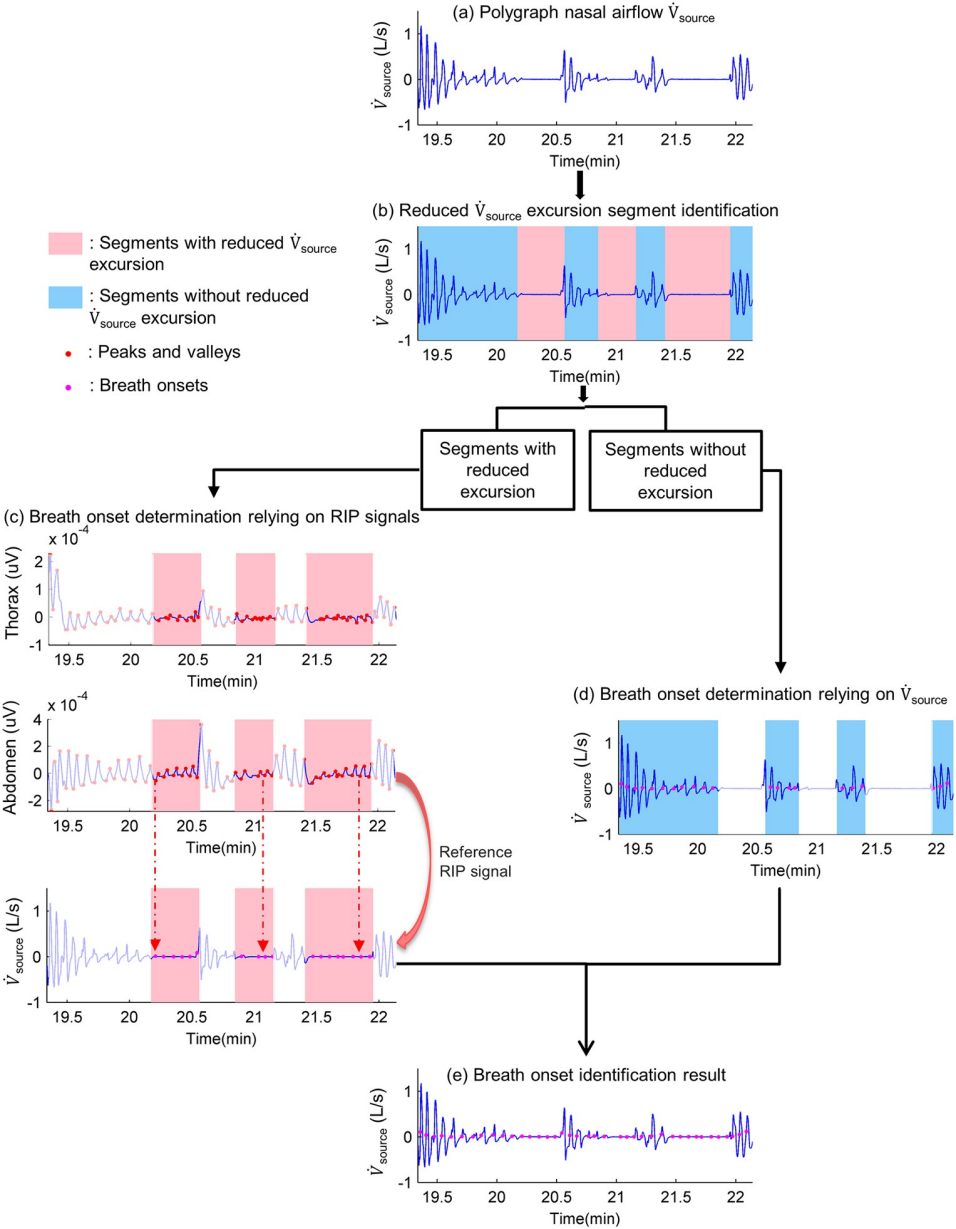
Identification of breath onset positions in polygraph nasal airflow. (a) Polygraph nasal airflow: an example of polygraph nasal airflow (${\dot{\text{V}}}_{\text{source}}$), of which we need to identify breath onset positions. (b) Reduced excursion segments identification: ${\dot{\text{V}}}_{\text{source}}$ is segmented in function of its excursion: periods with reduced excursion (pink shade) and periods without reduced excursion (blue shade). (c) Breath onset determination relying on Respiratory Inductance Plethysmography (RIP) signals: during periods with reduced ${\dot{\text{V}}}_{\text{source}}$ excursion, as breath onset positions are difficult to be directly determined from ${\dot{\text{V}}}_{\text{source}}$, the reference RIP signal’s valleys temporal coordinates are used to indicate the corresponding breath onset positions in ${\dot{\text{V}}}_{\text{source}}$. The choice between RIP thorax and RIP abdomen to be the reference RIP signal is determined by criterion of possessing a higher relative amplitude modulation compared to the other one. (d) Breath onset determination relying on ${\dot{\text{V}}}_{\text{source}}$: in segments without reduced ${\dot{\text{V}}}_{\text{source}}$ excursion, breathing onsets can be directly determined as the inflection point in ${\dot{\text{V}}}_{\text{source}}$ between expiratory minimal and inspiratory maximal values. (e) Breath onset identification result: all breath onsets in ${\dot{\text{V}}}_{\text{source}}$ are obtained by combining results from (c) and (d).

The proper identification of each breathing onset from ${\dot{\text{V}}}_{\text{source}}$ is mandatory to simulate corresponding respiratory cycles (Fig 5). As breathing onset positions are difficult to determine directly from ${\dot{\text{V}}}_{\text{source}}$ during highly-reduced breathing periods, it is necessary to apply a methodology to identify breathing onsets depending on the amplitudes of the nasal airflow excursion signal. Firstly, we calculated the excursion of ${\dot{\text{V}}}_{\text{source}}$. We detected inspiratory peaks and expiratory valleys in a three-minute moving window by using an automatic multiscale-based peak detection (AMPD) algorithm developed by Scholkmann *et al*. [22]. The 3-minute window was chosen in consideration of both computational time and algorithm performance. Indeed, the AMPD algorithm has an O(n^2^) complexity (n: signal length contained in a window), meaning that the smaller the window size is, the shorter the computational time will be. However, it should also contain enough breathing cycles so that the AMPD algorithm can capture the periodic pattern. In average, a 3-minute window contained about 45 (Ttot = 4s) to 72 (Ttot = 2.5s) breathing cycles, of which the amount was comparable to examples cited in Scholkmann et al’s paper [21]. Moreover, we also checked whether there were any missed cycle detections, by detecting oscillation around zero values with respect to amplitude threshold and a temporal threshold compatible with a respiratory cycle. These thresholds were established based on knowledge related to the mean amplitude and total respiratory cycle time (T_tot_) of previous respiratory cycles without obstruction. Thus, the upper envelope and lower envelope of ${\dot{\text{V}}}_{\text{source}}$ were obtained by interpolating peaks and valleys, respectively. Excursion was calculated as the difference between the upper envelope and lower envelope. Based on the excursion values, breathing onsets were then determined with two distinct methods. Considering periods with high excursion values, breathing onset positions were determined as the inflection point in ${\dot{\text{V}}}_{\text{source}}$ between expiratory minimal and inspiratory maximal values respecting the order of two consecutive cycles. However, our algorithm relied on RIP signals to infer breathing onset positions for low nasal airflow excursion periods.

Highly reduced excursion criteria were defined as a reduction of nasal airflow excursion equal or greater than 65% of the two-minute pre-event baseline as well as a duration greater than four seconds. This airflow reduction threshold was determined with reference to AASM apnea scoring rules. As nasal airflow was calculated by square-root transformation of nasal pressure, the nasal pressure reduction threshold (≥ 90%) used in apnea detection would correspond to a reduction greater than 66.7% in nasal airflow. We used a threshold of 65% very close to this. The duration criterion of at least four seconds was set up to allow for detecting any single obstructive or central apneic breathing cycle. A breathing cycle lasted about 3 seconds. Another 1 second was added in order to account for the time delay between the airflow valley of the last breath before reduced excursion and the start of the reduced excursion.

Considering highly-reduced excursion periods, the breathing onset cannot be identified on ${\dot{\text{V}}}_{\text{source}}$ by definition. Thus, we collected this information from the RIP signal. We determined for each period which RIP signal to refer to, namely thoracic or abdominal. This determination of the reference RIP signal was based on the following requirement: it need be the signal with a higher relative amplitude modulation during the reduced nasal airflow excursion period. Once we had decided upon the reference RIP signal, we used valley time coordinates in the reference RIP signal as the ones of breath onsets for the reduced nasal airflow excursion segments.

#### Step 4: Detection of apnea events and identification of significant breath attempts

Apnea events were detected according to AASM recommendations for apnea rules updated in 2012 [20]. The main signal used for apnea detection was ${\dot{\text{V}}}_{\text{source}}$. We set a maximal apnea event duration of three minutes to discriminate between reduced breathing and mouth breathing. Furthermore, apnea events detected by ${\dot{\text{V}}}_{\text{source}}$ were only considered if there was a simultaneous drop in the thermistance excursion signal or alternatively in the RIP flow excursion signal, which should be greater than 20% compared to the pre- and post-event baselines. For a given period, apneas were directly qualified as central apnea if there were no identified breathing attempts within the RIP signals. Moreover, we checked for the significance of the muscular effort indirectly represented in RIP signals for each breath attempt contained during the period of apnea. Significant breath attempts with collapsed upper airways, namely the obstructive apnea cycles, were then identified; otherwise, they were qualified as central due to a significant reduction of respiratory muscular effort modulation.

For each breath attempt, the significance threshold was set as 10% of the thoracic or abdominal RIP excursion baseline. The respiratory effort excursion of each breath attempt within an apnea was determined as the excursion value, whose time axis coordinate corresponds to this of the peak position in the RIP signal. The excursion baseline was determined as the within the one minute pre- and post-event excursion average.

#### Step 5: Detection and characterization of hypopnea events

Our algorithm detected hypopnea events according to the scoring rules of AASM updated in 2012 [20]. Similar to apnea detection, we mainly used ${\dot{\text{V}}}_{\text{source}}$ to detect hypopneas. Our algorithm also assessed RIP flow excursion or naso-buccal thermistance excursion. A descending tendency of that signal greater than or equal to 10% in comparison to the one-minute pre- and post-event baseline was required to exclude the possibility of mouth breathing, which could cause an amplitude drop in ${\dot{\text{V}}}_{\text{source}}$ as well. Additionally, to assure that this drop in nasal airflow excursion greater or equal to 30% was not due to sensor displacement, we also set a maximal event duration of three minutes.

Moreover, we followed the classification rules recommended by AASM [20] to classify hypopnea events as either obstructive or central hypopneas. Indeed, to consider an obstruction in the upper airway, one of the three following criteria was required: i) snoring, ii) thoracoabdominal paradoxical movements, or iii) flattened inspiratory airflow shape specific to obstruction.

In summary:

i) We evaluated the audio power difference between the inspiratory phase and expiratory phase to detect snoring.
ii) To identify the occurrence of thoracoabdominal paradox, the algorithm was based on the temporal closeness between peak and valley positions in RIP thorax and those in RIP abdomen. For example, during a given cycle with a thoracoabdominal paradox, the RIP thorax peak was supposed to be temporally closer to the valley rather than peak in RIP abdomen.
iii) We were inspired from the methodology proposed by Zhi *et al*. [23] to detect an inspiratory flow limitation in ${\dot{\text{V}}}_{\text{source}}$. Briefly, we trained a four-layer neural network (7*14*14*1) with seven features extracted from inspiratory airflow as inputs. The features were peak numbers, peak amplitude normalized by precedent 2-minute peak amplitude baseline, scooping index, kurtosis, deviation index, flattening index, skewness.

If none of these three criteria were detected, the candidate hypopnea event was considered as a central event.

#### Step 6: Calculation of digital inputs for integration into bench hardware

The active lung simulator is controlled through the simulated respiratory muscular effort pressure ΔP_mus_. The relationship between ΔP_mus_ and ${\dot{\text{V}}}_{\text{cc}}$ is modeled by Eq (1). ${\dot{\text{V}}}_{\text{cc}}$ was assigned directly to ${\dot{\text{V}}}_{\text{source}}$ for respiratory cycles included in normal breathing or in central respiratory events; ${\dot{\text{V}}}_{\text{cc}}$ was then estimated by a sinusoidal form, with an amplitude equal to the 2-minute pre-event baseline for respiratory cycles involved in obstructive events. Inspiratory and expiratory time of ${\dot{\text{V}}}_{\text{cc}}$ in obstructive cycles was calculated with respect to the temporality of ${\dot{\text{V}}}_{\text{source}}$ (for obstructive hypopneas) and the reference RIP signal (for obstructive apneas).

Due to technical limitations in our bench hardware, inspiratory and expiratory volumes needed to be equal for each cycle. Consequently, we equilibrated inspiratory and expiratory airflow with respect to their inspiratory peak and expiratory valley coordinates.

The resistance in Starling resistor is controlled by adjusting the sealed chamber pressure P_ch_. Here, the aim was to apply the appropriate P_ch_ to transform the amplitude of ${\dot{\text{V}}}_{\text{cc}}$ into the desired ${\dot{\text{V}}}_{\text{bench}}$ in order to simulate ${\dot{\text{V}}}_{\text{source}}$. Preliminary work allowed us to study the relationship between ${\dot{\text{V}}}_{\text{cc}}$ and the resulting flow after application of various P_ch_, that is to say various levels of obstruction. This relationship was studied respectively for inspiration and expiration. This work allowed us to calculate P_ch_ for each obstructive cycle as a function of ${\dot{\text{V}}}_{\text{cc}}$ and ${\dot{\text{V}}}_{\text{source}}$.

### Bench simulation performance evaluation

We compared mainly ${\dot{\text{V}}}_{\text{bench}}$ with ${\dot{\text{V}}}_{\text{source}}$ to evaluate our bench simulation performance with respect to two categories: 1. normal and hypopnea breathing; 2. apneas.

For normal and hypopnea breathing, we assessed the agreement between ${\dot{\text{V}}}_{\text{bench}}$ and ${\dot{\text{V}}}_{\text{source}}$ cycle-by-cycle in terms of relative peak-to-valley amplitude (A) and T_tot_ by means of linear regression analysis, histogram representation of bias, and Bland and Altman analysis [24]. We measured the similarity in airflow morphology between ${\dot{\text{V}}}_{\text{bench}}$ and ${\dot{\text{V}}}_{\text{source}}$ cycle-by-cycle by calculating a Pearson correlation coefficient (r) for each corresponding pair of cycles respectively in ${\dot{\text{V}}}_{\text{source}}$ and ${\dot{\text{V}}}_{\text{bench}}$. We assumed that the number of airflow samples in each respiratory cycle was ≥ 40 and that the distribution was normal. Then we calculated the mean ± one standard deviation (SD) of r following two separated groups of respiratory cycles: cycles respectively with obstruction and without obstruction in upper airways.

For apneas, we calculated sensitivity and Positive Predictive Value (PPV) to evaluate the correspondence between apneas detected in ${\dot{\text{V}}}_{\text{source}}$ and those in ${\dot{\text{V}}}_{\text{bench}}$. To assess apnea onset time agreement as well as apnea duration agreement between ${\dot{\text{V}}}_{\text{source}}$ and ${\dot{\text{V}}}_{\text{bench}}$, we evaluated apnea onset time differences by calculating its mean and SD, and performed linear regression and Bland and Altman analyses for apnea duration.

## Results

**Fig 6:**
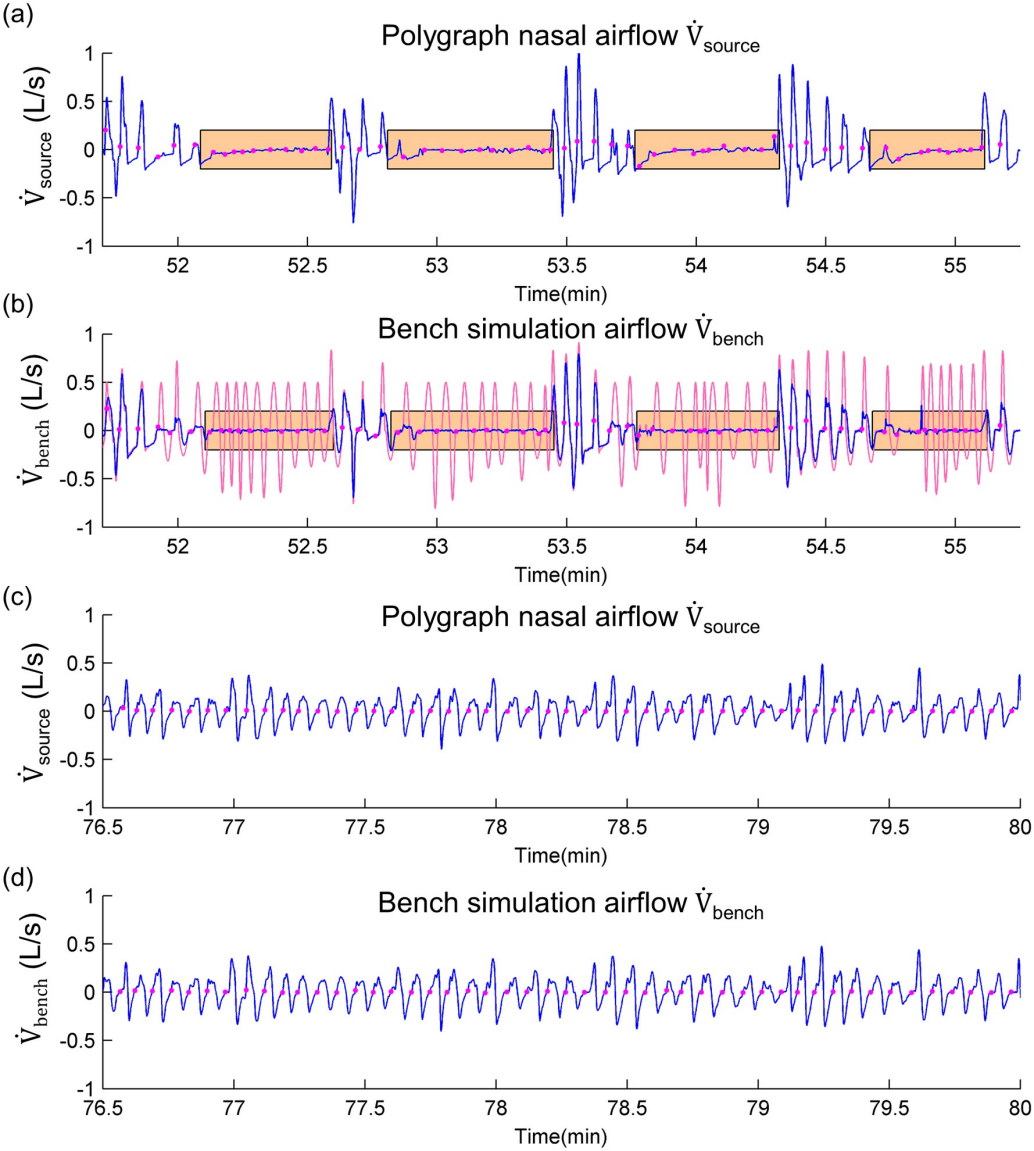
Illustration of bench simulation results: Polygraph nasal airflow versus bench airflow. (a) and (b): example of a breathing profile with obstructive apnea events marked in orange shade. The polygraph nasal airflow (${\dot{\text{V}}}_{\text{source}}$) and bench simulation airflow (${\dot{\text{V}}}_{\text{bench}}$) are respectively showed in (a) and (b) with blue curves. The red curve in (b) represents the estimated non-obstructed nasal airflow (${\dot{\text{V}}}_{\text{cc}}$), issuing from a central ventilation command. (c) and (d): example of a breathing profile without disordered breathing events. The ${\dot{\text{V}}}_{\text{source}}$ and ${\dot{\text{V}}}_{\text{bench}}$ are respectively showed in (c) and (d) with blue curves. The magenta points in all graphs represent the breath onsets.

Our algorithm allowed us to simulate a breathing profile from polygraph recordings by using a bench. Fig 6 shows two extracts of the bench simulations. The extracts describe respectively an obstructive SDB period and another breathing period without disordered breathing events. There is a graphical correspondence in signal amplitude and breathing events between the bench and the polygraph signals.

**Fig 7:**
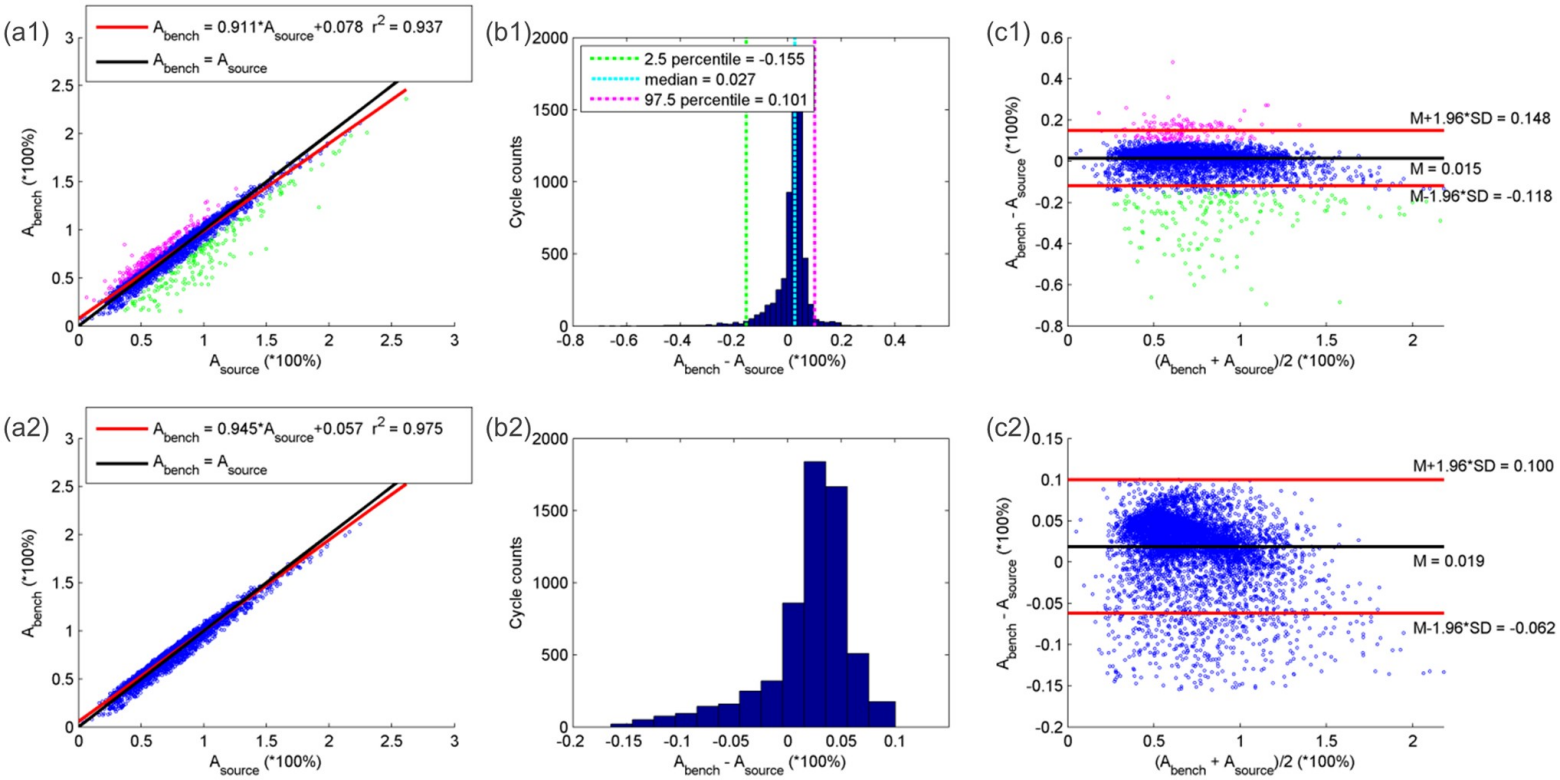
Linear regression, histogram, Bland and Altman plot analyzing similarity in relative amplitude between polygraph nasal airflow and bench airflow for 6,479 normal and hypopnea breathing cycles. (a1): Linear regression between polygraph estimated nasal airflow cycle relative amplitude (A_source_) and bench airflow cycle relative amplitude (A_bench_). Red line: the linear regression line. Black line: the identity line. Magenta points: breathing cycles with the difference between A_bench_ and A_source_ (A_bench_ − A_source_) greater than 97.5 percentile. Blue points: breathing cycles with A_bench_ − A_source_ between 2.5 percentile and 97.5 percentile. Green points: breathing cycles with A_bench_ − A_source_ lower than 2.5 percentile. (b1): histogram analysis of difference between A_bench_ and A_source_. Green cyan and magenta dashed line: their x-axis coordinates represent respectively 2.5 percentile (-15.5%), median (2.7%) and 97.5 percentile (10.1%) of A_bench_ − A_source_. (c1): Bland-Altman plot analyzing the agreement between A_bench_ and A_source_. Magenta, blue and greens points represent the same breathing cycles as described in graph (a1). Graphs (a2), (b2), (c2): the same analyses as in graphs (a1), (b1) and (c1) except that the cycles taken into account are those whose A_bench_ − A_source_ is between the 2.5 percentile and 97.5 percentile.

**Fig 8:**
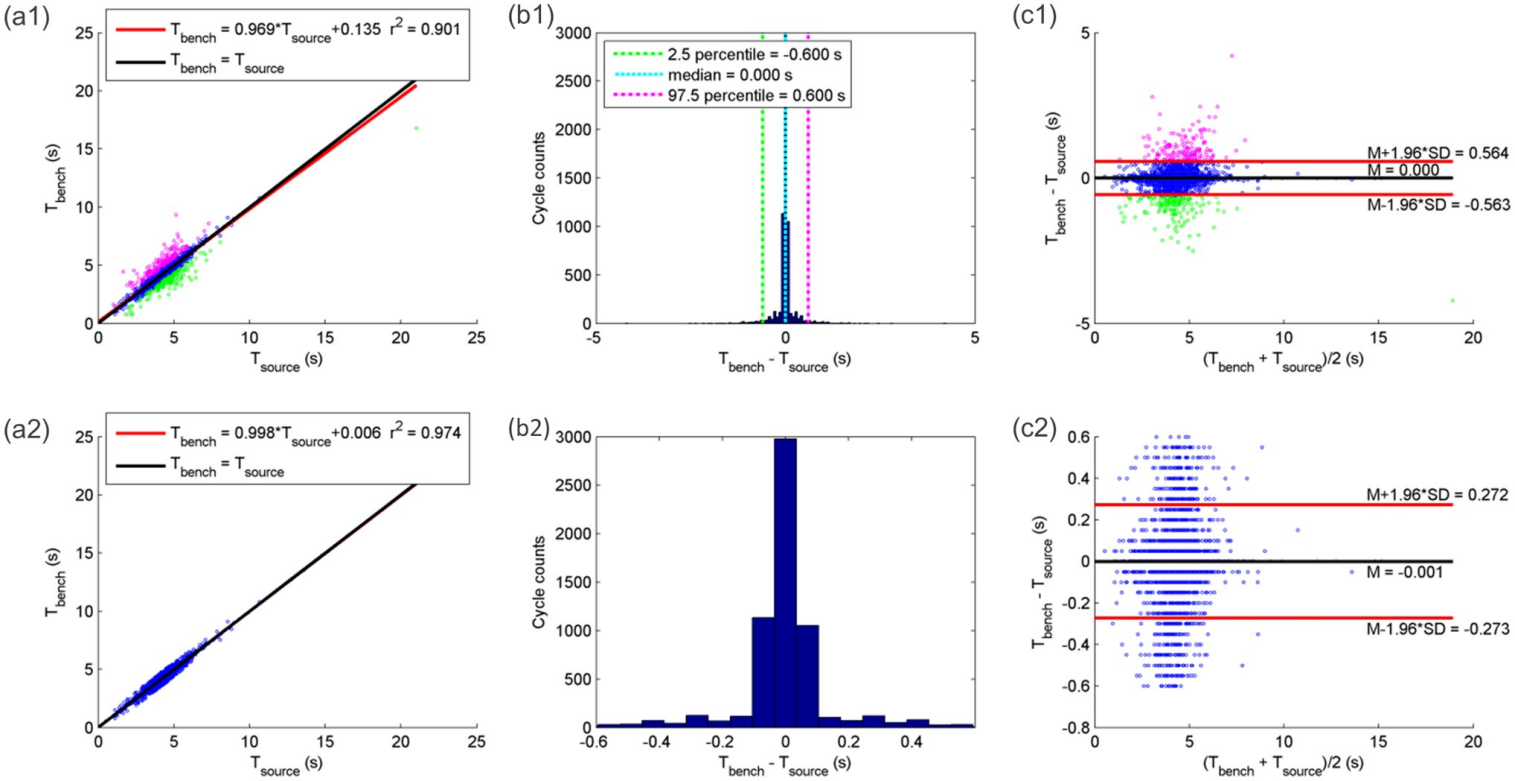
Linear regression, histogram, Bland and Altman plot analyzing similarity in total respiratory cycle time between polygraph nasal airflow and bench airflow for 6,479 normal and hypopnea breathing cycles. (a1): Linear regression between polygraph nasal airflow cycle time (T_source_) and bench airflow cycle time (T_bench_). Red line: the linear regression line. Black line: the identity line. Magenta points: breathing cycles with the difference between T_bench_ and T_source_ (T_bench_ − T_source_) is greater than 97.5 percentile. Blue points: breathing cycles with T_bench_ − T_source_ between the 2.5 percentile and 97.5 percentile. Green points: breathing cycles with T_bench_ − T_source_ lower than the 2.5 percentile. (b1): Histogram analysis of the difference between T_bench_ and T_source_. Green cyan and magenta dashed line: their x-axis coordinates represent respectively 2.5 percentile (-0.600 seconds), median (0 seconds) and 97.5 percentile (0.600 seconds) of T_bench_ − T_source_. (c1): Bland-Altman plot analyzing the agreement between T_bench_ and T_source_. Magenta, blue and greens points represent the same breathing cycles as described in graph (a1). Graphs (a2), (b2), (c2): the same analyses as in graphs (a1), (b1) and (c1), except that the cycles taken into account are those whose T_bench_ − T_source_ is between the 2.5 percentile and 97.5 percentile.

Concerning bench simulation performance for normal breathing (5,476 cycles) and hypopnea breathing (1,003 cycles), the average bias (M) in A between ${\dot{\text{V}}}_{\text{source}}$ and ${\dot{\text{V}}}_{\text{bench}}$ was 1.48% with a SD of 6.80% (Fig 7). Taking into account the 95% percent of cycles distributed around M, the SD of their biases decreased to 4.12%, whereas the M remained more or less unchanged (1.88%). The M in T_tot_ for each corresponding pair of respiratory cycles respectively in ${\dot{\text{V}}}_{\text{source}}$ and ${\dot{\text{V}}}_{\text{bench}}$ was approximately 0, with a SD equal to 0.288 seconds (Fig 8). Considering the 95% percent distributed around the M in T_tot_, the M ± one SD was -0.001 ± 0.139 seconds. The similarity assessed by Pearson correlation coefficient between each pair of respiratory cycles respectively in ${\dot{\text{V}}}_{\text{source}}$ and ${\dot{\text{V}}}_{\text{bench}}$ was 0.98 ± 0.08 considering all the normal and central hypopnea respiration cycles, and 0.87 ± 0.22 among all the obstructive hypopnea respiration cycles.

**Fig 9:**
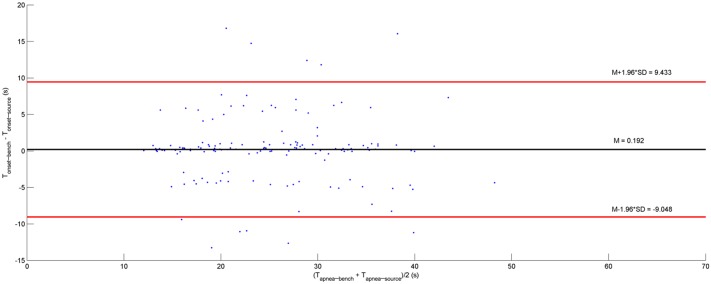
The relationship between apnea-onset time difference and apnea duration for apneas detected correspondingly in both bench airflow and polygraph nasal airflow (149 apneas). T_apnea-bench_: duration of apneas detected in **bench airflow** (${\dot{\text{V}}}_{\text{bench}}$). T_apnea-source_: duration of apneas detected in polygraph nasal airflow (${\dot{\text{V}}}_{\text{source}}$). T_onset-bench_: the apnea onset time of apneas detected in ${\dot{\text{V}}}_{\text{bench}}$. T_onset-source_: the apnea onset time of apneas detected in ${\dot{\text{V}}}_{\text{source}}$.

**Fig 10:**
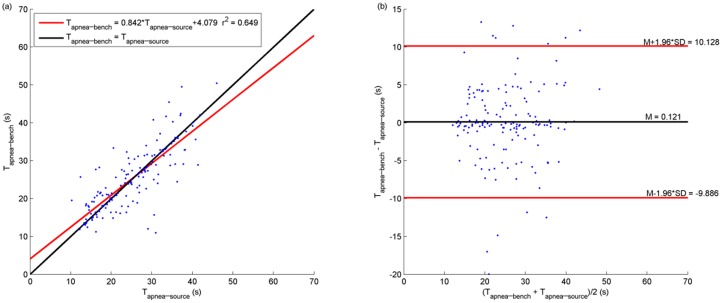
Linear regression, Bland and Altman analyses of apnea duration determined from respectively bench airflow and polygraph nasal airflow (149 apneas). Only the apneas detected both in bench airflow (${\dot{\text{V}}}_{\text{bench}}$) and in polygraph nasal airflow (${\dot{\text{V}}}_{\text{source}}$) are taken into account. (a): linear regression analysis between apnea duration determined from ${\dot{\text{V}}}_{\text{source}}$ (T_apnea-source_ in x-axis) and the one from ${\dot{\text{V}}}_{\text{bench}}$ (T_apnea-bench_ in y-axis). Red line: the linear regression line. Black line: the identity line. (b): Bland and Altman analysis of agreement between T_apnea-source_ and T_apnea-bench_.

As for apnea events, simulation sensitivity and PPV were respectively equal to 84.7% and 90.3% by taking into account 149 apneas occurring correspondingly both in ${\dot{\text{V}}}_{\text{source}}$ and ${\dot{\text{V}}}_{\text{bench}}$. Concerning the precision of apnea onset time, the average difference between apnea onset time in ${\dot{\text{V}}}_{\text{source}}$ and ${\dot{\text{V}}}_{\text{bench}}$ is equal to 0.19 seconds with a SD of 4.71 seconds (Fig 9). Concerning apnea event duration, the average bias is 0.12 seconds with a SD of 5.11 seconds, comparing the corresponding pair of apneas occurring in ${\dot{\text{V}}}_{\text{source}}$ and ${\dot{\text{V}}}_{\text{bench}}$ (Fig 10).

## Discussion

### Findings statement

Our study proposed a physiological method for developing a bench to test different APAP devices. By processing polygraph data, we derived the digital inputs required to instruct bench hardware, of which the active lung simulator was driven by ΔP_mus_, and a Starling resistor was regulated by P_ch_ to mimic a polygraph nasal breathing profile on the bench as similarly as possible. The bias existing in the airflow peak-valley amplitude can be partly explained by the limits of pressure regulators in terms of dynamic time response to a decreased P_ch_, corresponding to breathing cycles represented in green points in Fig 7. Moreover, this is also related to the incertitude in controlling the resistance effect in the Starling resistor.

Our bench seems to have an excellent performance in cycle period simulation and good similarity in terms of signal morphology between ${\dot{\text{V}}}_{\text{source}}$ and ${\dot{\text{V}}}_{\text{bench}}$, especially for respiratory cycles without obstruction in the upper airways. However, as for obstructive breathes, similarity was moderate. This can be explained by the fact that when increasing the resistance in the Starling resistor, we can no longer control the ${\dot{\text{V}}}_{\text{bench}}$ signal shape. We can only make sure that there are inspiratory flow limitation phenomena occurring among the obstructive breathing cycles and the amplitudes of these obstructive cycles are very similar to those in ${\dot{\text{V}}}_{\text{source}}$.

There was satisfactory sensitivity and a good PPV for apnea-event correspondence between ${\dot{\text{V}}}_{\text{source}}$ and ${\dot{\text{V}}}_{\text{bench}}$. There was some mismatch between bench and polygraph recordings for apnea onset and duration. These values are perfectible by optimizing the resistance incertitude in the Starling resistor, thus allowing for the desired reduction in airflow amplitude, as well as by attenuating the artifact in the airflow. Indeed, the airflow artifact was caused by the promptly collapsing rubber tube in the Starling resistor during the transition phase from breathing without obstruction to breathing with obstruction. This artifact was proportional to the level of obstruction. We did not calculate the specificity because we could consider that normal and hypopnea breathing periods account for the majority of polygraph recording time. The correspondence for normal and hypopnea breathing in ${\dot{\text{V}}}_{\text{source}}$ and ${\dot{\text{V}}}_{\text{bench}}$ should always be near perfect.

### Advantages of our new approach

Compared to previous published bench tests, our bench system is able to automatically reproduce an apneic patient nasal breathing profile from its polygraph recordings for both obstructive sleep apnea syndrome and central sleep apnea syndrome. This assures that the bench-simulated airflow contains the breath-to-breath variability showed in real patient airflow. Our bench can simulate an unlimited spectrum of disturbed breathing events issuing from apneic patients of various phenotypes. Moreover, our bench arranges the occurrence of different disordered breathing events in a physiological order. These depend on many factors related to patient characteristics such as age, gender, body mass index, craniofacial structure, as well as night-to-night variation like sleep stage, body position, alcohol or drug use, etc. Furthermore, the disordered breathing events of the obstructive mechanism simulated on our bench are capable of reacting to small steps of pressure change delivered by APAP devices by gradually increasing airflow amplitudes. This is a so-called closed loop.

### Limits of the bench

Only one Starling resistor was used in our study, whose geometrical and mechanical property was unique, such as in the collapsible tube’s ellipticity, wall stiffness, and upstream resistance at the onset of inspiratory flow limitation [25]. Thus, it may not represent the physical properties of all apneic patient upper airways. For the critical closing airway pressure, while it can be set to different values by varying P_ch_ in the Starling resistor, we do not have any information about a patient’s real critical closing airway pressure from the polygraph examination, nor regarding an effective treatment pressure. Accordingly, the critical closing airway pressure and the effective treatment pressure, which were specific to our test bench, could differ from that of patients. By simulating a patient breathing profile and testing it with different devices on our bench, we cannot determine the exact pressure range needed by a particular patient. However, according to the treatment performance that each device demonstrates and the pressure range used by each device, we can always recommend a suitable device for a particular patient, despite the fact that we cannot know the real treatment pressure range that the patient needs. Furthermore, our bench does not take into account patient physiological responses to previous treated or partially-treated disordered breathing events, like its ventilatory stability [26], which may influence the occurrence of upcoming disturbed breathing events.

### Future improvements

Concerning inspiratory flow limitations in the simulation method, there are two different modes in our bench according to whether the inspiratory flow limited cycle is contained in an obstructive disordered breathing event. If it is, the cycle is simulated with a partially closed rubber tube in the Starling resistor, and the ΔP_mus_ of this cycle is derived from ${\dot{\text{V}}}_{\text{cc}}$. In this case, the bench-generated limited flow shape may present a difference with the one in polygraph recordings. Otherwise, it is simulated by integrating the ΔP_mus_ derived directly from the ${\dot{\text{V}}}_{\text{source}}$, with a completely opened Starling resistor. This results in more similar inspiratory flow limitation morphology, however, without a true obstruction in the Starling resistor.

In the near future, we are considering implementing two other versions of algorithms on our bench, using two different methodologies (version I and version II) to simulate patient inspiratory flow limitation.

Version I: all cycles presenting an inspiratory flow limitation would be simulated as obstructive cycles with the rubber tube in Starling resistor partially collapsed;

Version II: all cycles presenting an inspiratory flow limitation would be simulated in the same manner as cycles of normal breathing or a central breathing event with the rubber tube in the Starling resistor fully open.

These two versions are complementary. With version I, we can learn to what extent the tested ventilation device is able to adapt the treatment pressure to an inspiratory flow limitation, and our bench will react to the pressure change delivered by the device in a closed loop. With version II, our bench would simulate the exact airflow shape of these cycles with an inspiratory flow limitation. In this way, we can learn whether the test device accurately recognizes such inspiratory flow limitations.

## Conclusion

Our new approach for APAP devices test bench overcomes previous existing constraints in simulating all kinds of breathing phenotypes in apneic patients by using a bench. This new physiological bench provides a more detailed characterization of different respiratory devices responses to a specific patient profile. It can serve as an aid tool for personalized therapy to facilitate device selection, option settings, etc. This work reproduces the breathing profile at night during sleep registered in polygraph. The next step would be to further validate this bench by integrating different patient polygraph recordings in order to evaluate inter-individual as well as intra-individual variabilities.
